# Two Ectopic Liver Lobes Discovered Incidentally at an Autopsy: A Case Report

**DOI:** 10.7759/cureus.52270

**Published:** 2024-01-14

**Authors:** Satoshi Sumida, Nobuo Satake, Koichi Tsuneyama

**Affiliations:** 1 Department of Pathology and Laboratory Medicine, Institute of Biomedical Sciences, Tokushima University Graduate School, Tokushima, JPN; 2 Department of Pathology, Ehime Prefectural Central Hospital, Matsuyama, JPN; 3 Department of Clinical Laboratory, Yoshinogawa Medical Center, Yoshinogawa, JPN

**Keywords:** ectopic liver lobe, liver development, anomaly, portal venopathy, autopsy

## Abstract

The ectopic liver lobe is a rare anomaly and is most frequently reported as a solitary mass. Herein, we report a case of multiple (two) ectopic liver lobes detected at an autopsy.

A Japanese man in his 70s died of an infectious disease associated with acquired immunodeficiency syndrome (AIDS). Autopsy revealed the incidental finding of two 1-cm masses, located anterior to the inferior vena cava. Both masses were composed of liver tissue and had internal microscopic structures resembling the porta hepatis, consisting of an outflow bile duct and blood vessels. The outflow bile duct appeared to be continuous with the common bile duct, but the connection point of the outflow vessel was unclear. The liver tissue showed fibrous thickening of the central veins and portal venopathy, including fibrosis in the portal area as well as narrowing and loss of the portal veins. There was no evidence of congestion, fibrosis, biliary stasis, or neoplasm.

The incidence of hepatocellular carcinoma is higher in the ectopic liver lobe than in the proper liver, presumably due to the abnormal circulation and bile excretion pathways. The patient also presented with portal venopathy; this suggests the presence of abnormal circulatory dynamics.

## Introduction

The ectopic liver lobe is a rare anatomic abnormality that occurs with a frequency of 0.24%-0.56% [1]. It is often asymptomatic and generally first noticed during surgery or at autopsy [2]. Most reported cases are solitary, and cases with multiple ectopic liver lobes are rare. Herein, we report a case of two 1-cm ectopic liver lobes located adjacent to each other and anterior to the inferior vena cava.

## Case presentation

Clinical course and autopsy findings

**Figure 1:**
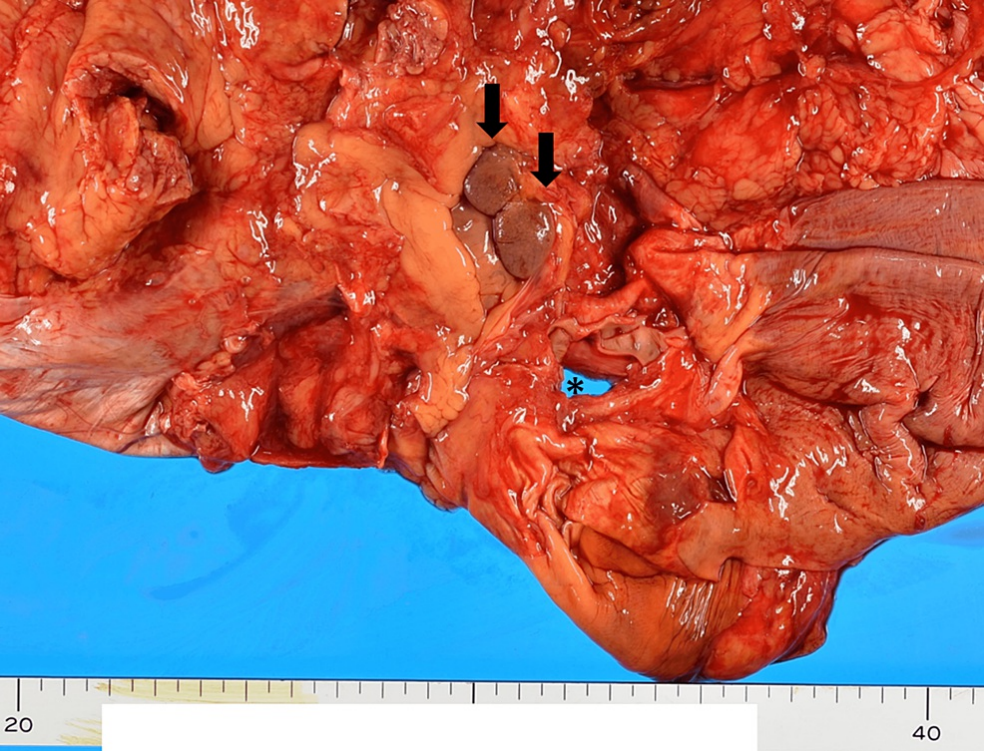
Macroscopic appearance of the ectopic liver lobes After the liver is dissected, the diaphragm is observed from the abdominal side. Two 1-cm brownish masses are arranged in juxtaposition (arrows); two masses were distinct. No continuity between the masses and diaphragm was detected. The asterisk indicates an esophageal hiatus.

A Japanese man in his 70s who had been diagnosed with oral candidiasis presented with dyspnea. Close examination revealed elevation of b-D-glucan and positivity of cytomegalovirus (CMV) antigen in serum, indicating a diagnosis of pneumocystis pneumonia and CMV infection. Multiple opportunistic infections led to suspicion of human immunodeficiency virus (HIV) infection, so further research was undergone. HIV infection was revealed by chemiluminescent immunoassay, and a diagnosis of acquired immunodeficiency syndrome (AIDS) was made; after the diagnosis of AIDS, serum HIV-1 RNA was detected (19,000 copies/mL). Imaging examinations of the abdomen performed at that time detected no obvious mass in the abdomen. Treatment for HIV infection was not performed; highly active antiretroviral therapy (HAART) or other antiretroviral treatment was not available due to the capacity of the institution, and his general condition was very poor for consultation with another institution. Despite treatment for opportunistic infection by trimethoprim-sulfamethoxazole and ganciclovir, the infectious diseases worsened, and the patient died approximately two weeks after referral. An autopsy was performed to elucidate the status of infectious diseases and therapeutic efficiency. The autopsy revealed that infectious diseases were progressive under the treatment, with additional findings of cryptococcosis and aspergillosis. At autopsy, two adjacent 1-cm masses were found near the inferior vena cava (Figure 1).

The masses had a smooth surface and color similar to that of the liver proper. No congestion, biliary stasis, fibrosis, or tumor was apparent from observation of the cut surface. The masses were separate from the liver and gallbladder, and no continuity was seen between them. Any outflow blood vessels or bile ducts were not detected grossly.

Pathological findings

**Figure 2:**
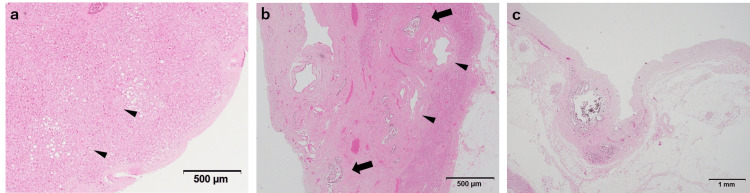
Architecture of the ectopic liver lobe (a) The ectopic liver lobe is covered with fibrous capsules. Arrowheads indicate Glisson's sheath. (b) An outflow bile duct (arrows) and medium-sized blood vessels (arrowheads) are seen, mimicking porta hepatis. (c) Additional sectioning of perihepatic tissue revealed a 1-mm duct with the appearance of a bile duct connecting the ectopic liver lobe with the common bile duct.

The surface of each mass was covered by a fibrous capsule, and each mass was composed of liver tissue consisting of hepatic lobules and Glisson’s sheath, which led to the diagnosis of an ectopic liver lobe (Figure 2, Panel a). Structures mimicking the porta hepatis, consisting of a bile duct and medium-sized blood vessels (thick-walled artery and dilated thin-walled vein), were also detected (Figure 2, Panel b). Additional investigation of the perihepatic tissue suggested continuity of the outflow bile duct with the common bile duct, but the continuity of the medium-sized blood vessels was unclear (Figure 2, Panel c).

**Figure 3:**
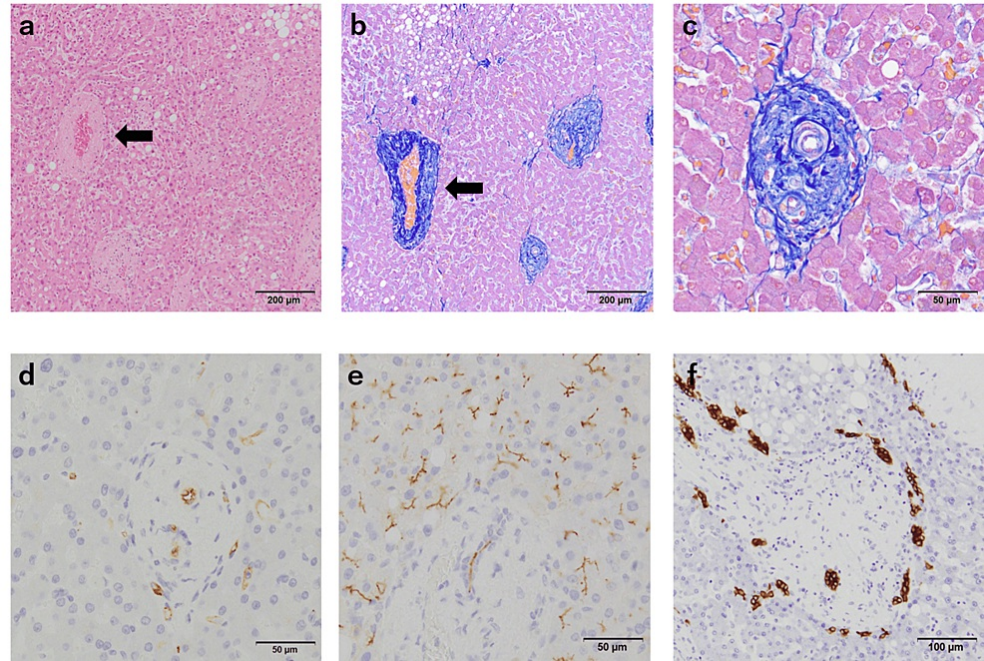
Histological features of the ectopic liver lobe (a,b) Central vein walls (arrows) are thickened by fibrosis (a: HE stain, b: Azan stain). (c) High-power view of Figure 3b. Portal fibrosis is prominent, and portal veins are unclear. (d) No capillarization of sinusoids is detected by immunohistochemistry (IHC) for CD34. (e) The formation of biliary canaliculi is confirmed by IHC for CD10. (f) IHC for cytokeratin 7. A ductular reaction is present.

The walls of central veins were thickened by fibrosis, with luminal narrowing (Figure 3, Panels a and b). Portal fibrosis was prominent and narrowing of portal veins was seen, mimicking portal venopathy (Figure 3, Panel c). The sinusoids were not dilated, and no capillarization of sinusoids was detected by immunohistochemistry (IHC) for CD34 (Figure 3, Panel d). There was no biliary stasis, and the formation of biliary canaliculi was confirmed by IHC for CD10 (Figure 3, Panel e), although the proliferation of bile ductules was observed, mimicking the ductal plate (Figure 3, Panel f).

**Figure 4:**
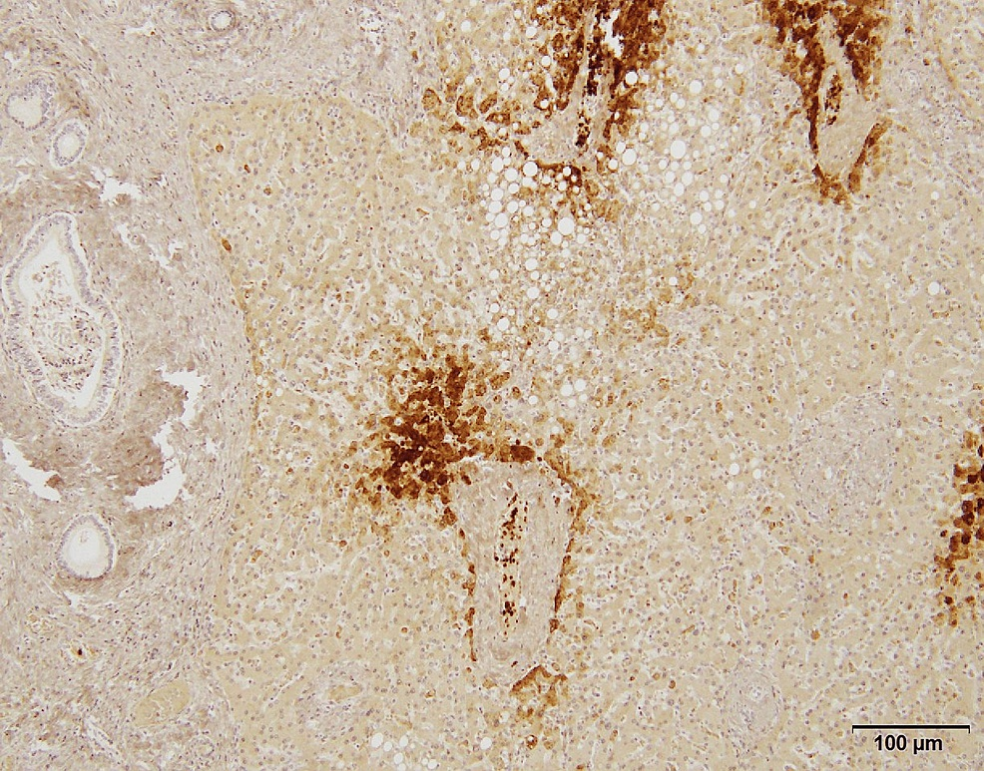
Immunohistochemistry for glutamine synthetase (GS) Central veins are surrounded by GS-positive hepatocytes as seen in normal liver tissue.

Glutamine synthetase (GS)-positive hepatocytes were distributed around the central veins as seen in normal liver tissue (Figure 4).

No remarkable change was detected in hepatocytes other than mild steatosis. The liver proper showed focal CMV infection, cryptococcal infection, and partial obstruction of central veins. There was no significant steatosis, hepatitis, or fibrosis. Liver lesions due to HIV were not detected either. No neoplastic lesion was detected either in the ectopic liver lobe or in the liver proper.

## Discussion

The ectopic liver lobe is a rare anomaly, as shown above. The most common site of ectopic liver lobe formation is the gallbladder; however, other abdominal sites have been reported, including the spleen, pancreas, adrenal glands, retroperitoneum, diaphragm, portal vein, and umbilical vein as well as thoracic sites such as the inferior vena cava, right atrium, lung and chest wall, and gluteal region [1,3-6]. In most cases, an ectopic liver lobe is a solitary lesion, and there are few reports of multiple ectopic liver lobes or ectopic liver lobe-derived neoplastic disease [4,7]. Collan et al. have classified ectopic liver lobe tissue into four categories: (i) accessory lobe: large, connected to the liver by a thin stalk; (ii) small accessory lobe: attached to the liver, weighing about 10-30 g; (iii) ectopic liver: not continuous with the liver and attached to adjacent organs such as the gallbladder or intra-abdominal ligaments; and (iv) microscopic ectopic liver: histologically identified on structures such as the gallbladder wall [8]. In the present case, two masses were not continuous with the liver, and biliary output was suggested to be the common bile duct. Thus, although vascular connections were not elucidated sufficiently, it seemed reasonable that two masses were classified as "ectopic liver."

Although multiple hypotheses have been proposed for the mechanism of ectopic liver lobe development depending on the site, explanations such as regression of the original connection between the accessory lobe and the main liver or aberrant migration or displacement of a portion of pars hepatica to other sites are widely accepted [9-11]. In the present case, two ectopic liver lobes were formed adjacent to each other in the vicinity of the inferior vena cava, in a location relatively close to the bile ducts. Continuity with the common bile duct was suspected, and the formation of biliary canaliculi in the hepatocytes by CD10 immunostaining suggested that a bile excretion pathway, or “biliary system for ectopic liver lobe,” had developed. In conclusion, displacement of a portion of pars hepatica, or pars cystica, was a possible mechanism for the development of an ectopic liver lobe in the present case.

Histologically, the ectopic liver lobe is described as showing histological features similar to normal liver tissue or liver proper with changes such as congestion, hemosiderosis, fatty infiltration, and fibrosis [2,10]. In the present case, the liver was considered normal except for cryptococcal and CMV infection as well as focal obstruction of central veins. However, the ectopic liver lobes showed an image resembling portal venopathy, different from that of the liver proper. In normal livers, blood returns from the hepatic vein to the inferior vena cava, whereas in ectopic liver lobes, the circulatory dynamics differ due to the abnormal position of the vein corresponding to the hepatic vein in the liver proper, resulting in portal venopathy or congestion.

The frequency of neoplastic change such as hepatocellular carcinoma is higher in the ectopic liver lobe than in the main body of the liver [3,12], and detection of neoplasia is most likely the most common means whereby the ectopic liver lobe is detected [7]. The reason for the higher frequency of tumorigenesis in the liver proper is probably because of the difference in circulatory dynamics and bile excretion function compared to the normal liver with associated oxidative and immune-modulatory stressors underlying this propensity [3,12]. In the present case, the presence of abnormal blood flow was suggested by histological features corresponding to portal venopathy, although tumorigenesis was not identified. The present findings may be a milestone for the elucidation of the relationship between abnormal blood flow and tumorigenesis in the ectopic liver lobe. Further study using cases with both abnormal blood flow and hepatocellular carcinoma is needed. Lastly, the liver and spleen are common sites of extramedullary hematopoiesis; this phenomenon was not observed in the ectopic liver lobes assessed as part of this case.

## Conclusions

We reported a relatively rare autopsy case of two adjacent ectopic liver lobes. Although the vascularity could not be sufficiently clarified, the histological features suggested a different circulatory system to that of a normal liver. Because the ectopic liver lobe is a rare entity that can occur at various anatomic sites, there are many cases of ectopic liver lobe arising outside the gallbladder in which the pathogenesis, circulatory dynamics, and bile excretion pathways have not been fully elucidated. The incidence of hepatocellular carcinoma is higher in the ectopic liver lobe than in the liver proper, presumably due to the abnormal circulation and bile excretion pathways. Further investigation based on the accumulation of cases is desirable.
